# Public mental health: An opportunity to support collaborative action

**DOI:** 10.1192/j.eurpsy.2025.60

**Published:** 2025-08-26

**Authors:** M. Destoop

**Affiliations:** 1 CAPRI, Antwerp University; 2 Multiversum, Antwerp, Belgium

## Abstract

**Abstract:**

Public mental health (PMH) involves a population-based approach to improve coverage, outcomes and coordination of PMH interventions to treat mental health conditions, to prevent associated burden, to prevent mental health conditions from arising, and promote mental well‐being and resilience. Collaborative care models involve population-based care teams sharing a defined group of patients tracked in a registry to ensure no one falls through the cracks. Practices track and reach out to patients who are not improving or who do not seek help (yet).

**Disclosure of Interest:**

None Declared

